# Comparative Transcriptomic Analysis Underlies the Differential Virulence of *Vibrio harveyi* and *Vibrio vulnificus* in American Eels (*Anguilla rostrata*)

**DOI:** 10.3390/ijms262411763

**Published:** 2025-12-05

**Authors:** Qiuhua Yang, Guanghua Sun, Sijia Hong, Qi Lin, Jinjin Yang, Songlin Guo

**Affiliations:** 1 Fisheries College, Jimei University, Xiamen 361021, China; qhyang1314@163.com (Q.Y.); sunshine19991230@163.com (G.S.); elsa123456_8@163.com (S.H.); 17746313318@163.com (J.Y.); 2 Key Laboratory of Cultivation and High-Value Utilization of Marine Organisms in Fujian, Fisheries Research Institute of Fujian Province, Xiamen 361013, China; xmqlin@sina.com; 3 Engineering Research Center of the Modern Industry Technology for Eel, Ministry of Education of PRC, Xiamen 361021, China; 4 State Key Laboratory of Mariculture Breeding, Fisheries College, Jimei University, Xiamen 361021, China

**Keywords:** *Vibrio harveyi*, *Vibrio vulnificus*, *Anguilla rostrata*, pathology, differentially expressed genes, differentially alternative splicing genes

## Abstract

*Vibrio harveyi* (Vh) and *Vibrio vulnificus* (Vv) are major bacterial pathogens affecting farmed eels, but their comparative virulence mechanisms remain poorly characterized. This study combined histopathology and transcriptomic profiling to investigate organ-specific damage and host responses in American eels (*Anguilla rostrata*, 20 g per fish, for a total of 60 fish) following experimental infection with LD_50_ doses of Vh (strain HA_1, 7.5 × 10^4^ CFU/fish) and Vv (strain FJ_4, 5.0 × 10^5^ CFU/fish). Tissue samples from liver, kidney, and spleen were collected at 0, 36, and 60 h post-infection (hpi). Histopathological analysis revealed distinct injury patterns: Vh induced severe hepatic edema and necrosis, whereas Vv caused vacuolar degeneration and vascular congestion in the liver. In the kidney, Vv triggered acute necrosis and vacuolization by 36 hpi, while Vh-induced renal damage was delayed until 60 hpi. Transcriptomic analysis of spleen tissue identified 4779 and 1215 differentially expressed genes (DEGs) in the Vh_36 vs. Vv_36 and Vh_60 vs. Vv_60 comparisons, respectively. Functional enrichment analysis associated these DEGs with 109 Gene Ontology (GO) terms—mainly catalytic activity, biological regulation, and binding—and 51 KEGG pathways, including “tuberculosis” and “pathways in cancer”. Differential alternative splicing (DAS) analysis further uncovered 1579 and 1214 DAS events originating from 12,482 and 12,316 splicing genes in the two comparisons. These were enriched in GO categories such as “binding”, “cellular process”, and “cell part”, as well as KEGG pathways related to “signal transduction”, “infectious diseases”, and “immune system.” Protein–protein interaction network analysis identified 119 cross-DAS-encoded proteins, including 8 that were predicted as key regulators of virulence differences. In summary, this work presents the first integrative study comparing the pathogenicity and host transcriptional dynamics of Vh and Vv in American eels, providing new molecular insights into their distinct virulence strategies.

## 1. Introduction

The genus *Vibrio* comprises a group of common aquatic bacterial pathogens with broad pathogenicity across diverse host species. Their virulence is influenced by factors such as host immunity and environmental conditions [1,2]. Vibriosis, caused by various *Vibrio* species, occurs globally and results in substantial economic losses in aquaculture [3,4,5]. Among these, *Vibrio harveyi* and *Vibrio vulnificus* are Gram-negative, facultatively anaerobic, opportunistic pathogens widely distributed in aquatic environments, capable of infecting a variety of aquatic animals and humans [6,7]. Infected fish often present with skin ulcers, systemic internal hemorrhage, and abdominal distension [8,9,10]. Clinical signs such as respiratory distress, anorexia, and abnormal excretion are also commonly observed [11,12,13]. Histopathological examination reveals hepatocyte necrosis, pallor of the liver, and hemorrhages in the spleen and kidney [9,14]. The disease progresses rapidly, often developing into extensive tissue necrosis, leading to mass mortality within seven days.

The pathogenesis of *V. harveyi* and *V. vulnificus* involves multiple virulence factors, such as adhesins, toxins (including enterotoxins, cytotoxins, hemolysins, endotoxins, and exotoxins), proteases, lipases, iron acquisition systems, capsules, biofilms, secretion systems, hemagglutinins, and flagella [15,16,17,18]. Adhesins facilitate tissue attachment and colonization, while type III and/or type VI secretion systems (T3SS, T6SS) promote invasion by delivering effector proteins into host cells [19,20,21,22]. In recent years, *V. harveyi* and *V. vulnificus* have been identified as common pathogens in farmed eels. These infections affect American, European, and Japanese eels in aquaculture systems [3,4,10,14,23]. Previous studies have documented the pathogenicity of these two species in eels [24], with one report indicating that *V. harveyi* exhibits higher virulence than *V. vulnificus*, as evidenced by a lower LD_50_ value in American eels after a 14-day challenge.

The American eel (*Anguilla rostrata*) is a catadromous fish species of significant economic and ecological importance. Characterized by its snake-like body shape, brownish-black dorsal side, and tan-yellow ventral side, this species exhibits broad salinity tolerance, enabling it to transition freely between freshwater and marine environments. It also has a wide range of thermal adaptability, with an optimal growth temperature of 20–28 °C [25]. Due to the decline in traditional aquaculture species such as the Japanese eel, the American eel has become an important alternative in aquaculture. Its advantages include strong environmental adaptability, high disease resistance, and a relatively stable supply of juveniles [26]. This species is well-suited for intensive farming systems and performs particularly well in recirculating aquaculture systems, achieving high survival rates [27]. However, after nearly a decade of large-scale cultivation in China, disease outbreaks have become increasingly frequent in farming American eels, with pathogens such as *V. harveyi* and *V. vulnificus* posing significant threats [3,28].

However, the mechanisms underlying the differential virulence between *V. harveyi* and *V. vulnificus* in American eels remain poorly understood. Therefore, investigating the pathogenic differences between *V. harveyi* HA_1 and *V. vulnificus* FJ_4—strains isolated from diseased American eels—is crucial for understanding the diseases they cause. In this study, American eels were challenged with LD_50_ doses of each pathogen to compare histopathological manifestations and host transcriptional responses via RNA-seq. The findings provide new insights into the virulence mechanisms of these economically important bacterial pathogens in infected fish.

## 2. Results

### 2.1. Comparative Histopathology of Liver, Kidney, and Spleen

**Figure 1:**
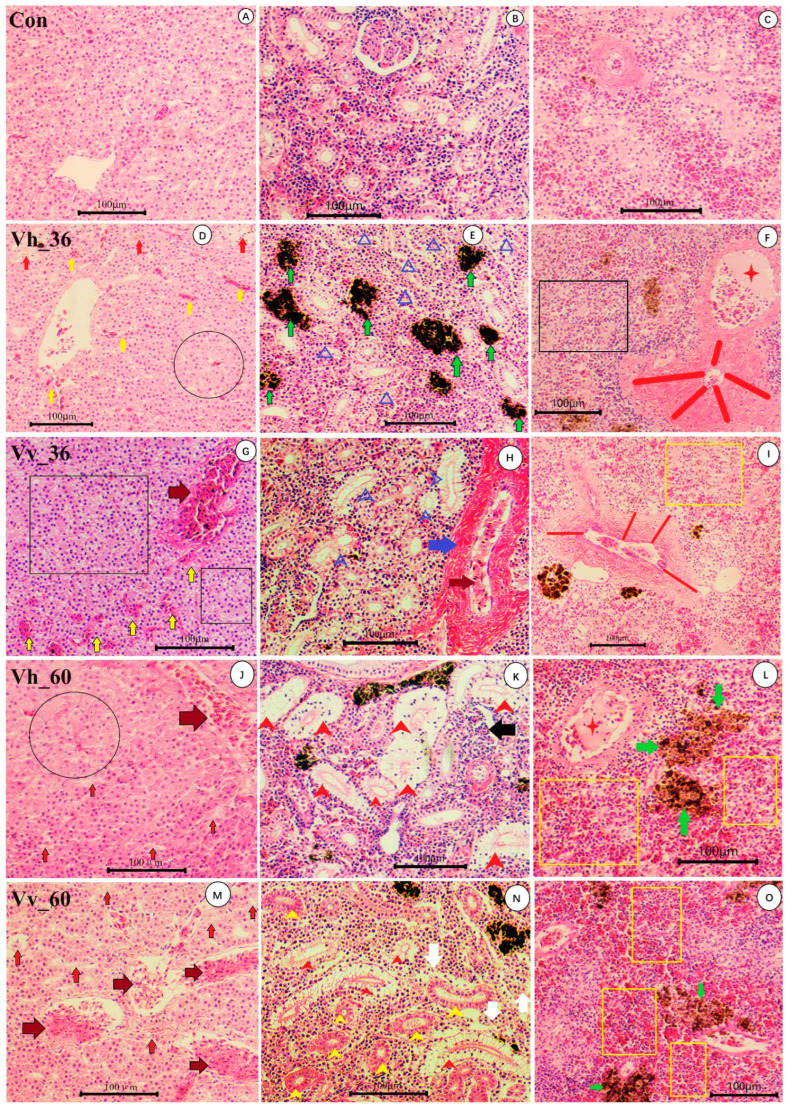
Pathological changes in liver (**D**,**G**,**J**,**M**), kidney (**E**,**H**,**K**,**N**) and spleen (**F**,**I**,**L**,**O**) post 36 h (Vh_36, Vv_36) and 60 h (Vv_60, Vh_60) infection of the *V. harveyi* HA_1 and *V. vulnificus* FJ_4 in American eel compared to the PBS injection eel (Con). “Vh” and “Vv” indicate *V. harveyi* HA_1 and *V. vulnificus* FJ_4, respectively. (**A**–**C**) indicate normal liver, kidney and spleen; (**D**) hemorrhage between liver tissues (yellow arrows), severe hepatocyte edema (circle), hepatocyte necrosis (red arrows); (**G**) congestion in vessels and increased red blood cells (deep red arrow), hemorrhage between liver tissues (yellow arrows), mild vacuolar degeneration of hepatocytes (square frame); (**J**) congestion in hepatic vein (deep red arrow), severe hepatocyte edema (circle), necrosis of hepatocytes (red arrow); (**M**) congestion in hepatic venules and capillaries (deep red arrow), necrosis of hepatocyte (red arrow). (**E**) Atrophy and granular degeneration of renal tubular epithelial cells (blue triangle), enlargement and increased number of phagocytic cell centers (green arrow); (**H**) thickening of hepatic artery walls, fibrous degeneration (blue arrow), intravascular congestion (deep red arrow), necrosis of some renal tubular epithelial cells (blue short headed arrow); (**K**) severe necrosis and vacuolization of renal tubular epithelial cells (red short headed arrow), glomerular capillary atrophy (black arrow), (**N**) granular degeneration of renal tubular epithelial cells (yellow short headed arrow), vacuolar degeneration of renal tubular epithelial cells (red short headed arrow), necrosis and disappearance of interstitial cells (white arrow). (**F**) Thickening of splenic arteriole walls, fibrous degeneration (red line), congested and thrombosis in splenic arterioles (four-pointed star), atrophy of splenic parenchymal cell (square frame); (**I**) thickening of splenic arteriole walls, fibrous degeneration (red line), enlargement of red pulp area (yellow frame); (**L**) increased red pulp and decreased white pulp (yellow frame), thrombosis in splenic arterioles (four-pointed star), increased central melanin engulfing cells (green arrow); (**O**) increased red pulp and decreased white pulp (yellow frame), increased central melanin engulfing cells (green arrow).

Prior to infection, the hepatic structure was normal, with hepatocytes arranged in cord-like patterns around a hepatic vein of typical size (Figure 1A). At 36 h post infection (hpi), eels infected with *V. harveyi* HA_1 exhibited hemorrhage between liver tissues, severe hepatocyte edema, and focal hepatocyte necrosis (Figure 1D). In contrast, *V. vulnificus* FJ_4 infection resulted in vascular congestion, intertissue hemorrhage, and mild vacuolar degeneration of hepatocytes (Figure 1G). By 60 hpi, *V. harveyi*-infected eels showed congestion in the hepatic vein, pronounced hepatocyte edema, and necrosis (Figure 1J), whereas *V. vulnificus*-infected eels displayed congestion in hepatic venules and capillaries, along with hepatocyte necrosis (Figure 1M).

In normal kidney tissue, the renal tubular epithelial cells are arranged in an orderly and structurally intact manner, and the glomerular capsule space is clear with continuous flattened parietal epithelial cells (Figure 1B). At 36 hpi, *V. harveyi* HA_1 infection led to atrophy and granular degeneration of renal tubular epithelial cells, as well as enlargement and increased numbers of phagocytic cell centers (Figure 1E). *V. vulnificus* FJ_4 infection resulted in thickening and fibrous degeneration of hepatic artery walls, intravascular congestion, and necrosis of some renal tubular epithelial cells (Figure 1H). By 60 hpi, *V. harveyi*-infected eels exhibited severe necrosis and vacuolization of renal tubular epithelial cells, along with glomerular capillary atrophy (Figure 1K). In comparison, *V. vulnificus*-infected eels showed granular and vacuolar degeneration of renal tubular epithelial cells, accompanied by necrosis and loss of interstitial cells (Figure 1N).

Uninfected eels displayed normal splenic architecture with distinct red and white pulp and numerous red blood cells visible in splenic arterioles (Figure 1C). At 36 hpi, *V. harveyi* HA_1 infection induced fibrous degeneration with thickening of splenic arteriole walls, congestion, thrombosis (indicated by four-pointed star), and atrophy of splenic parenchymal cells (Figure 1F). *V. vulnificus* FJ_4 infection also caused fibrous degeneration and arteriole wall thickening, along with an expanded red pulp area due to hemorrhage (Figure 1I). By 60 hpi, both infection groups showed increased red pulp, reduced white pulp, and numerically central melanin-engulfing cells. Thrombosis was notably present in the splenic arterioles of *V. harveyi*-infected eels (Figure 1L), whereas in *V. vulnificus*-infected eels, although similar pulp changes were observed, thrombosis was less prominent (Figure 1O).

### 2.2. Sequencing Data and Mapping Rate

Compared to the genomic DNA of *Aa*, the RNA reads of three control samples showed that the raw and clean reads were from 4.595 to 5.087 G and from 4.523 to 5.015 G, but those of the 12 infected samples were from 4.375 to 7.178 G and from 4.317 to 6.911 G (Table 1). Also, the sequencing error rate of 15 samples was lower than or equal to 2.67 in ten thousand; in all 15 sequences, the base error rate of more than 97.26 and 92.69 fragments was less than 1% (Q 20) and 1‰ (Q 30). The GC content of 3 control samples was a little lower than that of the 12 infected samples. The clean RNA reads of three control samples showed that the mapping rate of the total reads was a little more than 83%, and that of the 12 infected samples was from 83.291% to 87.30%. Moreover, the uniquely mapping rate in 12 infected samples was also higher than 3 control samples, with 82.20% and 80.54% of the average mapping rate, respectively (Table 2).

### 2.3. Gene Expression, Venn Analysis, Correlation and Clustering Between Samples

**Figure 2:**
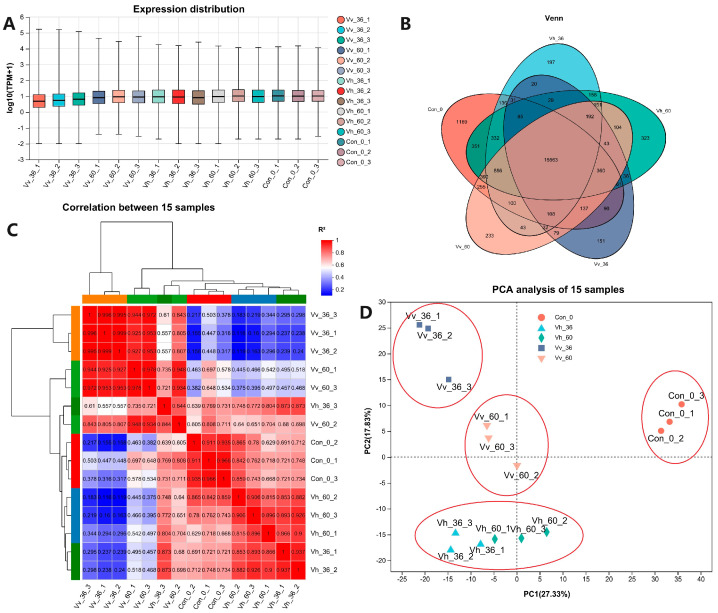
The expression distribution (**A**), Venn map (**B**), correlation (**C**) and PCA (**D**) between 15 samples based on all expressed genes (tpm ≥ 1).

Gene expression levels across the 15 samples showed an average of approximately 10 TPM (Transcripts Per Million). Notably, three samples from the Vv_36 group exhibited markedly lower expression levels compared to this average (Figure 2A). Venn analysis revealed 15,563 commonly expressed genes (TPM ≥ 1.0) across all groups. The Con_0 group contained 1169 uniquely expressed genes, a number substantially higher than that observed in the other four experimental groups (Figure 2B). Correlation analysis based on genes with TPM ≥ 1 clustered the 15 samples into six distinct branches: Branch 1 comprised the three Vv_36 samples; Branch 2 included Vh_60_1 and Vv_60_3; Branch 3 contained Vv_60_2 and Vh_36_3; Branch 4 consisted of the three control (Con) samples; Branch 5 grouped Vh_60_2 and Vh_60_3; and Branch 6 combined Vh_60_1, Vh_36_1, and Vh_36_2 (Figure 2C). Principal component analysis (PCA) performed on all expressed genes (TPM ≥ 1) further separated the samples into four clusters. Notably, all six samples infected with *V. harveyi* HA_1 grouped closely together (Figure 2D).

### 2.4. Differential Expressed Genes (DEGs): Venn and Heatmap Analysis of DEGs

**Figure 3:**
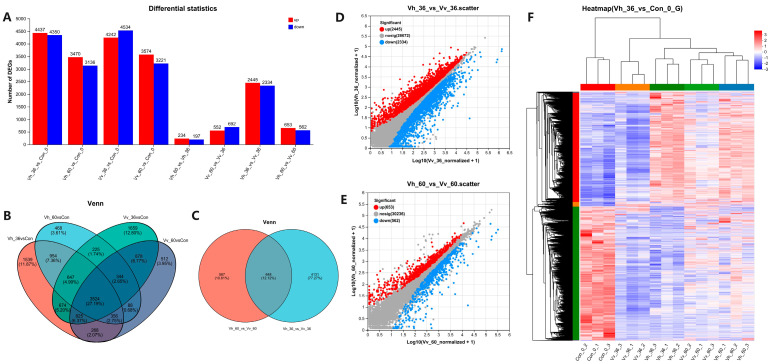
Column of up or down-regulated differentially expression genes (DEGs) in 8 comparisons (**A**), Venn map of DEGs in comparisons of 4 infection groups vs. Con (**B**), Venn map of 2 comparisons of Vh_36 vs. Vv_36 and Vh_60 vs. Vv_60 (**C**), up or down-regulated DEGs in 2 comparisons of Vh_36 vs. Vv_36 (**D**), Vh_60 vs. Vv_60 (**E**), and heat map (**F**) of 15 samples clustered by 8787 DEGs of Vh_36 vs. Con. (**D**,**E**) The x and y-axis value of a specific point is the logarithmic conversion value of the gene expression in control and processed sample, respectively. The red dots in the figure represent significantly up-regulated genes, the blue dots represent significantly down-regulated genes.

The numbers of up- and down-regulated DEGs in 8 comparisons were as follows: 4437/4350, 3470/3136, 4242/4534, 3574/3221, 234/197, 552/692, 2445/2334, and 653/562, respectively (Figure 3A). Venn analysis revealed 3524 common DEGs shared among the four infection-vs-control comparisons (Figure 3B). Additionally, 648 common DEGs were identified between the two cross-species comparisons (Vh_36 vs. Vv_36 and Vh_60 vs. Vv_60; Figure 3C). To display up- and down-regulated DEGs, the scatter plots for Vh_36 vs. Vv_36 (Figure 3D) and Vh_60 vs. Vv_60 (Figure 3E) plotted logarithmically transformed gene expression values on both axes. Hierarchical clustering based on the 8787 DEGs from the Vh_36 vs. Con comparison showed clear separation, with the three biological replicates from each of the five experimental groups clustering together distinctly (Figure 3F).

### 2.5. GO and KEGG Enrichment Analysis

**Figure 4:**
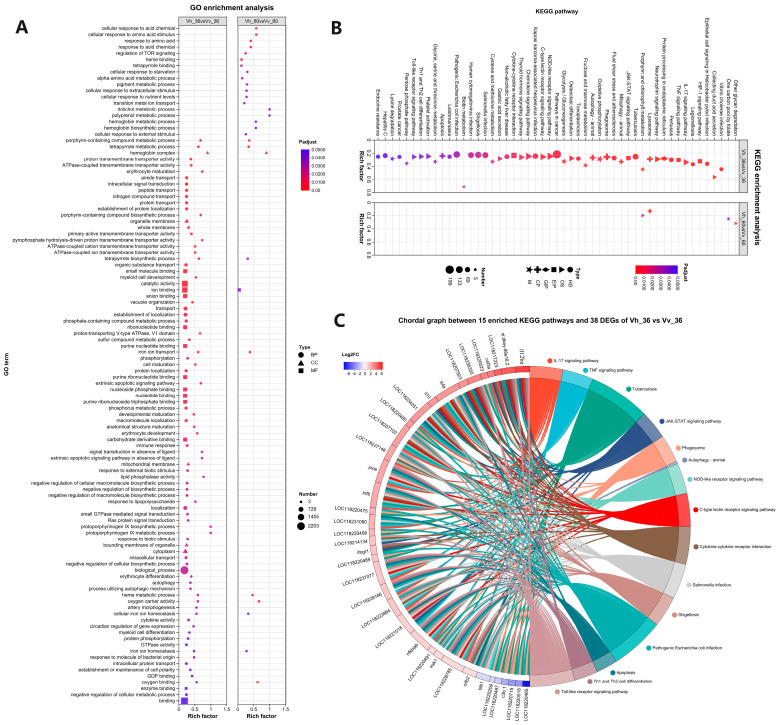
The 91 and 29 differential enrichment GO terms (**A**) and the 173 and 4 differential enrichment KEGG pathways (**B**) in two comparisons of Vh_36 vs. Vv_36 and Vh_60 vs. Vv_60 as well as the Chordal graph between 15 enriched KEGG pathways and 38 DEGs in Vh_36 vs. Vv_36 (**C**). (**A**) The type of BP, CC and MF indicates Biological Process, Cellular Component and Molecular Function; (**B**) the type of M, OS, HD, CP, GIP and EIP indicate Metabolism, Organismal Systems, Human Diseases, Cellular Processes, Genetic Information Processing and Environmental Information Processing in that order. (**A**,**B**) The vertical axis indicates the GO terms or KEGG pathways, and the horizontal axis indicates the rich factor ratio of regulated/background genes; the size of the dots indicates the number of genes, and the color of the dots indicate *p* adjust values.

GO analysis revealed 109 enriched terms across both comparisons. The Vh_36 vs. Vv_36 comparison had 91 terms, while Vh_60 vs. Vv_60 had 29, with 11 shared terms related to iron, heme, and oxygen processes (Figure 4A). The Vh_36 vs. Vv_36 comparison also featured 32 GO terms contained over 100 DEGs, and over 1000 DEGs in high-level terms like “biological process”, “binding”, and “catalytic activity”, whereas the Vh_60 vs. Vv_60 comparison had only one term with over 100 DEGs (Supplementary Table S1). KEGG analysis identified 51 enriched pathways. The Vh_36 vs. Vv_36 comparison had enriched 49 pathways, including “Pathways in cancer”, “Pathogenic Escherichia coli infection” and “Shigellosis” with over 100 DEGs. In contrast, Vh_60 vs. Vv_60 had only four enriched pathways, with 2 unique (other glycan degradation, one carbon pool by folate, Supplementary Table S2) and 2 common in the two comparisons (Figure 4B). Of 38 immune-related genes and 15 enriched pathways illustrated by a chord diagram in the Vh_36 vs. Vv_36 comparison (Figure 4C, Supplementary Table S3), 12 were up-regulated and at least linked to 7 pathways. Key genes, including *il*-1, *il*-6, *nf-κ*B, *ap*-1, and *tnf*, were highly involved, participating in 10 to 11 different pathways (Table 3).

### 2.6. Validation of qRT-PCR for RNA_Seq

**Figure 5:**
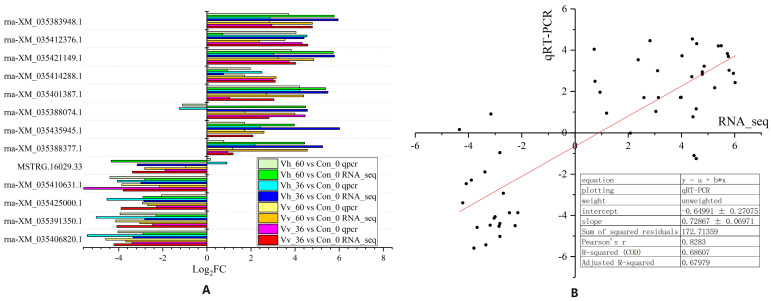
qRT-PCR vs. RNA-seq comparison of 13 transcripts expression levels by Colum (**A**) and Linear fit (**B**) in the spleen of American eels between 4 comparisons of Vv_36 vs. Con, Vv_60 vs. Con, Vh_36 vs. Con and Vh_60 vs. Con.

Of the 13 transcripts selected for validation, the fold-change values for 11 were consistent between qRT-PCR and RNA-seq across all four comparisons (Vv_36 vs. Con, Vv_60 vs. Con, Vh_36 vs. Con, and Vh_60 vs. Con). However, the regulation trends (up- or down-) of two transcripts (XM_035388074.1 and MSTRG.16029.33) observed by RNA-seq disagreed with those from qRT-PCR in the Vh_60 vs. Con and Vh_36 vs. Con comparisons (Figure 5A). A linear fit of the expression values for these transcripts revealed a strong correlation between the two methods, with a coefficient (slope) of 0.729 and a Pearson’s correlation coefficient of 0.828 (Figure 5B).

### 2.7. The Differential Alternative Splicing Genes (DASs) Analysis

**Figure 6:**
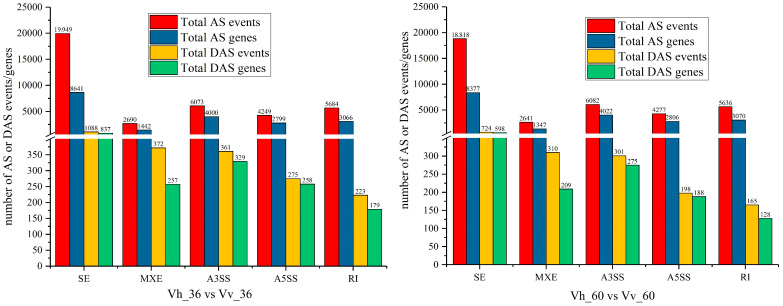
Number of the alternative splicing (AS) genes and events of 5 different AS types in two compares of Vh_36 vs. Vv_36 and Vh_60 vs. Vv_60. SE, IR, MXE, A5SS and A3SS indicate exon skipping, intron retention, exon mutual exclusion, variable 5′ splice site and variable 3′ splice site in that order.

The number of AS events was, on average, 3.10 and 3.04 times greater than the number of AS genes, respectively. From a total of 12,482 and 12,316 AS genes, only 1579 and 1214 were identified as significantly differential (DASs, FDR < 0.05). The average number of DAS events was 1.46 and 1.39 times the number of DAS genes in the two comparisons (Table 4). Five types of AS and DAS events were identified: exon skipping (SE), intron retention (IR), mutually exclusive exons (MXE), alternative 5′ splice sites (A5SS), and alternative 3′ splice sites (A3SS). The abundance of AS events and DAS genes across these five types was similar between the Vh_36 vs. Vv_36 and Vh_60 vs. Vv_60 comparisons (Figure 6) following challenge with *V. harveyi* or *V. vulnificus* in American eels (*Anguilla rostrata*).

### 2.8. The Results of the AS Verification

**Figure 7:**
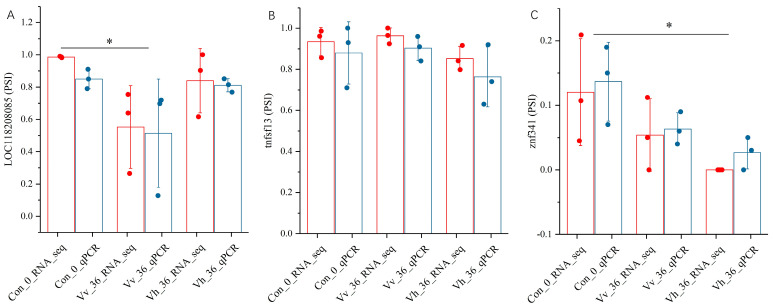
qRT-PCR vs. RNA-seq comparison of 3 skipped exon isoforms named *loc118208085* (**A**), *tnfsf13* (**B**) and *znf341* (**C**) in the spleen of American eels between 3 groups of Con_0, Vv_36 and Vh_36. The red dots represent qRT-PCR data and the blue dots represent RNA-seq data. Error bars indicate standard deviation (S.D). An asterisk (*) marks statistically significant differences (*p* < 0.05) between the groups when assessed by both the PSI values derived from qPCR and RNA-seq methods.

Infection with *V. vulnificus* for 36 h led to a significant down-regulation of the isoform of the *loc118208185* gene containing the skipped exon. Similarly, a notable decrease in the exon-containing isoform of the *znf341* gene was observed following 36 h of infection with *V. harveyi*. In contrast, the exon-skipping pattern of the *tnfsf13* gene showed no significant change after infection with either *Vibrio* strain, with only a slight down-regulation of the exon-containing isoform detected in the *V. harveyi*-infected group (Figure 7).

### 2.9. Annotation and Enrichment Analysis of DASs, DEGs, and Overlapping DAS-DEGs

**Figure 8:**
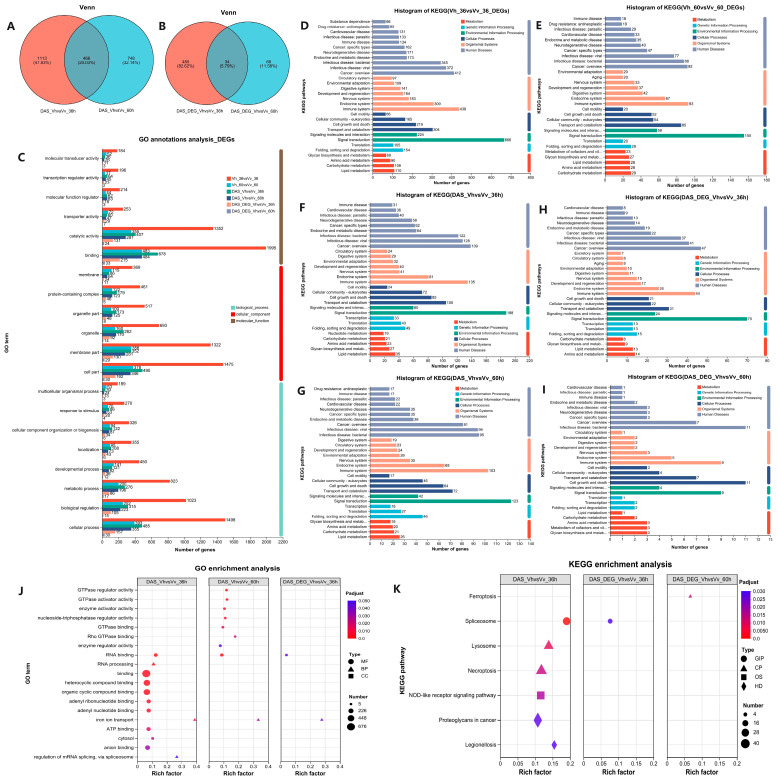
Venn maps between DAS genes (**A**) and between DAS genes cross DEGs (**B**) of Vh_36 vs. Vv_36 (**C**) and Vh_60 vs. Vv_60 (**D**); GO annotations of 6 gene sets (**C**); KEGG histogram of 5038 (**D**) and 1264 (**E**) DEGs, 1579 (**F**) and 1214 (**G**) DAS genes, and 519 (**H**) and 102 (**I**) DAS genes cross DEGs in the comparison of Vh_36 vs. Vv_36 and Vh_60 vs. Vv_60; the 12, 9 and 3 enriched GO terms (**J**) and the 6, 1 and 1 enriched KEGG pathways (**K**) in three comparisons. (**C**) The vertical axis indicates the GO terms and the horizontal axis indicates the number of DEGs; (**D**–**I**) the vertical axis indicates KEGG pathways, and the horizontal axis indicates the number of DEGs. (**J**) The type of BP, CC and MF indicates Biological Process, Cellular Component and Molecular Function; (**K**) the type of OS, HD, CP, and GIP indicate Organismal Systems, Human Diseases, Cellular Processes and Genetic Information Processing in that order. (**J**,**K**) The vertical axis indicates the GO terms or KEGG pathways, and the horizontal axis indicates the rich factor ratio of regulated/background genes; the size of the dots indicates the number of genes, and the color of the dots indicate *p* adjust values.

The Venn diagram shows that 466 genes with DAS were common to both comparisons (Figure 8A). When analyzing genes that were both differentially spliced and expressed (DAS-DEGs), the Vh_36 vs. Vv_36 comparison had 519, the Vh_60 Vv_60 had 102, and 34 overlapped (Figure 8B). Most of these genes were associated with fundamental functions like binding, cellular processes, and catalytic activity (Figure 8C). They were also predominantly enriched in key pathways such as “Signal transduction”, “Immune system” and “Infectious disease” (Figure 8D–I). The Vh_36 vs. Vv_36 comparison for DASs was the most active, with 12 significantly enriched GO terms, including substantial gene representation in “binding” (676 genes) and related molecular functions (Supplementary Table S4). In contrast, the Vh_60 vs. Vv_60 comparison and the overlapping DAS-DEGs of Vh_36 vs. Vv_36 showed far fewer enrichments (9 and 2 terms, respectively, Figure 8J). This pattern was mirrored in the KEGG pathway analysis. The Vh_36 DAS set was enriched in 6 pathways, with the top hits being “Proteoglycans in cancer” (40 DASs), “NOD-like receptor signaling pathway” (38), and “Necroptosis” (36), highlighting key roles in immune signaling and cell death (Supplementary Table S5). In contrast, the other two gene sets each had only a single enriched pathway (Figure 8K).

### 2.10. Protein–Protein Interaction Network of DAS Gene-Encoded Proteins

**Figure 9:**
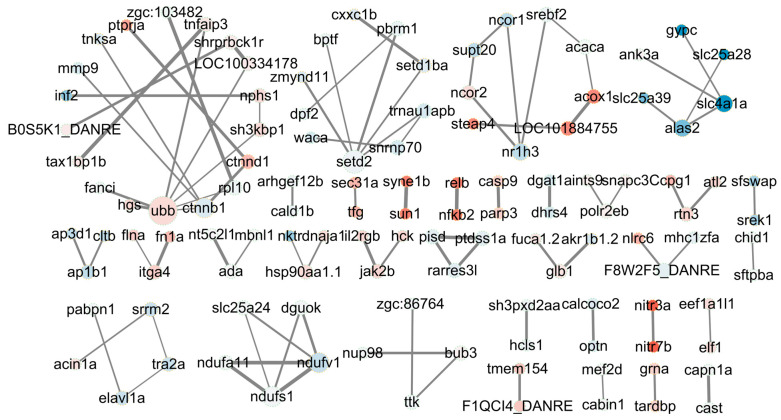
The 1–7 degree interaction networks between proteins expressed by 119 out of 466 cross DASs from 2 comparisons of DAS_VhvsVv_36h and DAS_VhvsVv_60h. The size of the DASs area indicates the degrees from 1 to 7; red background indicates up-regulation and blue background indicates down-regulation of the gene expression in the comparison of Vh_36 vs. Vv_36; the shade of the color indicates the degree of down and up regulations (log_2_FC = −4.08 to 1.69). The yellow edges of DASs indicate that these DASs are DEGs (padj < 0.05) and the green edges of DASs indicate these DASs are not DEGs (padj ≥ 0.05); the wide of the edges indicates the combined scores (from 0.70 to 0.99) between two proteins encoded by DASs.

Protein–protein interaction (PPI) analysis was performed on the 466 common DASs of Vh_36 vs. Vv_36_DAS and Vh_60 vs. Vv_60_DAS, revealing interactions between 119 of the encoded proteins (Supplementary Table S6). These 119 proteins formed 33 networks with a total of 91 interaction degrees (Figure 9). Although only 43 and 9 of these DASs were also identified as DEGs in the two respective comparisons, their alternative splicing was significant (FDR < 0.05). Notably, the proteins encoded by *ubb*, *setd2*, *ctnnb1*, *ndufs1*, and *ndufv1* were hub nodes, directly interacting with 7, 5, 4, 4, and 4 other proteins, respectively. The proteins encoded by *nr1h3*, *alas2*, and *slc4a1a* each interacted with 3 other proteins.

## 3. Discussion

Our previous studies identified *V. harveyi* HA_1 and *V. vulnificus* FJ_4 as two common bacterial pathogens affecting American eels. The reported median lethal doses (LD_50_) for these pathogens in American eels are 3.7 × 10^3^ CFU/g and 2.5 × 10^4^ CFU/g body weight, respectively [24]. In this study, we employed the LD_50_ dose to establish an infection, as it represents a threshold that affects 50% of the population, providing a balanced and controlled model for investigating pathogen-host interactions. This approach enables a preliminary exploration of the dynamics between host and pathogen under moderate conditions. We therefore investigated the causes of their differing virulence by examining pathological changes and conducting RNA-seq analysis on eels infected with the LD_50_ dose.

### 3.1. Pathological Changes and Differences in Virulence

In European eels, *V. harveyi* and *V. vulnificus* primarily cause necrosis of parenchymal cells in the liver and kidney, respectively [3,29]. However, infection with *V. vulnificus* and *V. harveyi* in this study induces distinct and severe pathologies in American eels (Figure 1). In the liver, the pathogens cause different forms of damage: *V. vulnificus* leads to hepatocyte vacuolar degeneration and vascular congestion, while *V. harveyi* triggers severe hepatocyte edema and necrosis. The necrosis from *V. harveyi* is likely driven by an acute inflammatory response (Figure 4C), mediated by up-regulated DEGs that promote the release of pro-inflammatory cytokines (e.g., TNF-α/β, IL-1β, IL-6 in Table 3), increasing vascular permeability and causing tissue damage [30,31]. In contrast, the pathology from *V. vulnificus* may result from direct bacterial toxins and the host’s inflammatory response, potentially elicited by proteins encoded by a unique set of 878 DEGs [32,33]. This pathology is likely elicited by proteins encoded by a subset of the 878 DEGs unique to the Vv_36 vs. Con_0 and Vv_60 vs. Con_0 comparisons (Figure 3B). These pathological changes contribute to liver dysfunction and can be life-threatening.

In the kidney, both pathogens cause necrosis of renal tubular epithelial cells by 60 hpi. However, *V. vulnificus* acts more rapidly, inducing acute necrosis as early as 36 hpi, attributed to inflammatory mediators, direct toxin cytotoxicity, and systemic hypoxic injury [34,35]. Conversely, *V. harveyi* infection results in serious vacuolization of renal cells at 60 hpi, preceded by enlarged phagocytic centers. This vacuolization may stem from direct toxin effects, a sustained inflammatory response, immune-mediated injury, or metabolic disturbances [3,36,37,38,39,40,41].

Splenic pathology also reveals key differences. Both pathogens cause red pulp enlargement, a sign of extramedullary hematopoiesis supported by the enrichment of GO terms related to heme metabolism and oxygen transport (Figure 4A), but this occurs earlier with V. vulnificus (36 hpi vs. 60 hpi) [42,43,44]. Uniquely, *V. harveyi* infection leads to parenchymal atrophy and arteriolar thrombosis. These severe lesions may arise from multiple mechanisms, including direct tissue invasion, toxin-induced damage, disseminated intravascular coagulation (DIC), septic emboli, immune-mediated vasculitis, hemodynamic instability, and the effects of lipopolysaccharide (LPS) [3,9,45,46,47,48,49,50]. These mechanisms illustrate the complex interplay between bacterial infection, inflammation, endothelial dysfunction, and thrombosis in the pathogenesis of splenic parenchymal atrophy and arteriolar thrombosis.

At the molecular level, both species utilize toxins and enzymes for pathogenicity, but differ in their strategies. *V. vulnificus* relies on exotoxins and extracellular enzymes (e.g., proteases, lipases) to cause cell lysis and necrosis, thriving in warm marine environments and posing a significant threat to immunocompromised fish [51,52]. *V. harveyi* also employs exotoxins and hemolysins to disrupt host cells, but is a ubiquitous pathogen capable of surviving in both freshwater and seawater, causing widespread economic losses in aquaculture by infecting diverse species [53,54,55].

### 3.2. Analysis of DEGs and Virulence Differences

Owing to the lack of a comprehensive gene annotation file for American eels, we utilized the European eel genome as a reference for our RNA-seq analysis. The high average alignment rate of ~85% (Table 2) across all samples confirmed its suitability for this study [56]. Global gene expression, measured in TPM, was notably suppressed in the *V. vulnificus* (Vv_36) group (Figure 2A), a finding consistent with the control group having a substantially higher number of uniquely expressed genes than any infected group (Figure 2B) [57].

Principal Component Analysis (PCA) showed that *V. harveyi*-infected samples formed a tight cluster, indicating a uniform host response. In contrast, *V. vulnificus*-infected samples separated into two distinct groups, suggesting a more variable and dynamic infection process (Figure 2D). Despite these differences, the total number of DEGs induced by each pathogen was similar at both time points, indicating that virulence disparity is not a simple function of DEG quantity [58]. A direct comparison between pathogens revealed a sharp decrease in DEGs over time (from 4779 at 36 hpi to 1215 at 60 hpi) (Figure 3A), underscoring that early, strain-specific responses are key. Venn analysis identified 954 and 878 unique DEGs for *V. harveyi* and *V. vulnificus*, respectively (Figure 3B), which likely underlie their distinct pathogenic mechanisms. Hierarchical clustering of all 15 samples showed clear segregation by group (Figure 3F), demonstrating fundamentally distinct gene expression profiles driven by the host’s response to each pathogen [59].

Gene Ontology (GO) enrichment revealed 11 common terms related to iron homeostasis and heme metabolism, indicating that both pathogens induce host response to hemolysis and hypoxia [60,61,62]. However, significant functional differences were evident in the acute phase (Vh_36 vs. Vv_36), with over 1000 DEGs in broad categories like ‘catalytic activity’, suggesting these nuances contribute to differential virulence [63]. KEGG pathway analysis further highlighted differences in critical processes, with pathways such as “Pathways in cancer” and “Pathogenic *E. coli* infection” being highly enriched (Figure 4B) [64,65]. Fifteen immune-related pathways were differentially regulated, primarily by 12 up-regulated DEGs encoding key immune proteins like TNF-α, interleukins, and chemokines (Figure 4C, Table 3) [66,67,68]. Key pathways of “Tuberculosis” and “Pathways in cancer” are interconnected through shared mechanisms of immune evasion, chronic inflammation [69], and central cytokine signaling [70].

QRT-PCR validation of 13 transcripts showed general consistency with the RNA-seq data in this study (Figure 5). Minor discrepancies for two transcripts are likely attributable to different transcript isoforms, such as alternatively spliced variants. Therefore, qPCR targets specific coding sequences providing a more comprehensive view of total gene expression [58,71].

### 3.3. Analysis of DAS Genes and Virulence Differences

Alternative splicing (AS) significantly contributes to proteomic diversity and functional regulation. Among over 12,400 and 12,300 AS genes analyzed, only 1579 and 1214 were differentially alternatively spliced (DAS) in the Vh_36 vs. Vv_36 and Vh_60 vs. Vv_60 comparisons, respectively (Table 4). This indicates that while infection induces widespread AS, only a specific subset is regulated in a strain-dependent manner. The strong concordance between qPCR and RNA-seq data validating skipped exon (SE) isoforms confirmed the reliability of our AS analysis (Figure 7) [72].

A key finding was the clear uncoupling of differential splicing from differential gene expression. At 60 hpi, despite a low number of DEGs (1215), there were 1214 DAS genes, with only 102 genes overlapping both categories (Figure 8B). This demonstrates that analyzing AS is essential for a complete understanding of virulence mechanisms, as gene expression changes alone are insufficient to explain the distinct LD_50_ values [73].

Functionally, the DAS genes were predominantly annotated to critical cellular processes like ‘binding’, ‘catalytic activity’, and ‘biological regulation’ (Figure 8C) [74]. KEGG analysis further confirmed their relevance, showing significant enrichment in “Signal transduction”, “Immune system”, and “Infectious disease” pathways (Figure 8D–I) [75]. DAS genes in the Vh_36 vs. Vv_36 comparison were highly enriched in ‘binding’ (Figure 8J), which are involved in transcription regulation, signal transduction, and metal catalysis [76,77,78]. Specific pathways enriched in DAS genes from the Vh_36 vs. Vv_36 comparison, such as “NOD-like receptor signaling”, “Necroptosis”, and the “Spliceosome”, directly point to mechanisms underlying the observed pathological differences (Figure 8K) [46,79,80]. For instance, “Necroptosis” enrichment suggests a different regulated cell death mechanism may be at play compared to the necrosis seen histologically.

A protein–protein interaction network constructed from 119 central DAS genes revealed key hub proteins—such as ubb, setd2, and ctnnb1—known to be regulators of inflammation (Figure 9) [81,82,83,84,85]. The fact that less than 40% of these central DAS genes were also DEGs, yet the infections have 50% mortality rates, strongly supports the conclusion that splicing changes are a major independent factor driving differential virulence.

In summary, this integrated analysis reveals that the differential pathologies caused by *V. harveyi* and *V. vulnificus* are driven not only by differences in gene expression, but also by strain-specific alterations in alternative splicing. These splicing events modulate key immune and cell death pathways, providing a novel molecular basis for their differing virulence.

## 4. Materials and Methods

The animal study protocol of this study was approved by the Ethics Committee of Jimei University (Approval Number: JMU2022030020; Approval Date: 16 March 2022), Xiamen, China.

### 4.1. Bacteria Strain and American Eels

*Vibrio harveyi* HA_1 and *V. vulnificus* FJ_4 were isolated from the ascites of American eels, as identified in our previous study [3,23]. A cohort of 60 healthy American eels, with an average body weight of 20 g, was procured from an eel farm situated in Fuqing, Fujian province. These eels were temporarily housed within a laboratory aquarium at a density of 20 eels per tank (200 L capacity) from 7 September to 5 October in 2023. After a 14-day acclimatization period, the water conditions were maintained as follows: temperature at 26–28 °C, pH between 6.6 and 7.3, ammonia nitrogen at 0.3–0.7 mg/L, nitrite at 0.17–0.23 mg/L, and dissolved oxygen at 8–12 mg/L, with continuous aeration provided by an oxygen pump. During this period, the eels were fed once daily, and the tank water was refreshed after a lapse of 2 h post-feeding. Prior to the *V. harveyi* and *V. vulnificus* infection, all eels were subjected to a 1-day fasting regimen.

### 4.2. Experimental Infection and Sampling

The *V. harveyi* and *V. vulnificus* were prepared using Tryptone soya broth (TSB), and the bacterial cells were concentrated to 1.5 × 10^6^ cfu/mL and 1.0 × 10^7^ cfu/mL after being diluted by PBS. Sixty eels were randomly divided into PBS injection (Con group), *V. harveyi* (Vh group) infection and *V. vulnificus* (Vv group) infection groups. Then, 3 groups of eels were infected by intraperitoneally received the PBS, LD_50_ dose of *V. harveyi* (7.5 × 10^4^ cfu/fish) and *V. vulnificus* (5.0 × 10^5^ cfu/fish) for 14 days post the infections [24]. As both groups of infected eels began to show obvious symptoms at 36 h post-infection (hpi) and some eels began to die at 60 hpi. Therefore, after 36 and 60 hpi, respectively, liver, kidney and spleen from 6 eels (30 mg/L Eugenol, 5 min before sampling) of every group were collected. For each eel collected from the 2 time points, spleens, livers and kidneys from 3 eels were fixed in 10% neutral formalin for paraffin section and these organs from other 3 eels were stored in RNA protect reagent at −70 °C for RNA extraction. For hygienic disposal of experimental fish and bacterial strain after the experiment, both were processed using the high-pressure steam sterilization method (115 °C, 15 min).

### 4.3. Preparation of Paraffin Sections

Samples fixed in neutral formalin were dehydrated using gradient alcohol, followed by transparentization in xylene and embedding in paraffin. After cutting into 5–7 μm sections, the sections were stained with H & E, and then dehydrated, transparentized. The outcome of the process was the acquisition of photographic evidence documenting the pathological alterations in the liver, kidney, and spleen of the examined eels.

### 4.4. Transcriptome Analysis Via RNA-Seq

RNA sequencing was performed on samples from the PBS control group (Con_0) and from American eels at 36 and 60 hpi with *V. harveyi* (Vh_36, Vh_60) or *V. vulnificus* (Vv_36, Vv_60). All sample processing, library construction, and sequencing were conducted by Majorbio (Shanghai, China). The procedure included the following steps: total RNA quality control, double-stranded cDNA synthesis, cDNA purification, end repair, A-tailing and adapter ligation, size selection, second-strand digestion, PCR amplification, and final library qualification before sequencing on an Illumina NovaSeq 6000 platform (San Diego, CA, USA). Sequencing quality was assessed based on error rate and Q-score statistics. Clean reads were aligned to the *Anguilla anguilla* reference genome (assembly accession: GCF_013347855.1) using Bowtie2. Gene expression levels were quantified with RSEM. The distribution of reads across exonic, intronic, and intergenic regions was analyzed based on alignment results. Transcript assembly and quantification were performed using StringTie v2.1.4 [86]. Subsequently, all transcripts were merged with Cuffmerge v2.0.2 [87], retaining only those longer than 200 nt and with unambiguous strand orientation. Known transcripts were functionally annotated based on the *A. anguilla* annotation file (.gff). For novel transcripts, coding potential was assessed using CPC2, Pfam 36.0, and CNCI 2.0 [88]. Those predicted to encode proteins were designated as novel mRNAs.

### 4.5. Analysis of Gene Expression and Differentially Expressed Genes

In this study, gene expression levels were quantified using Transcripts Per Kilobase per Million (TPM), and genes or mRNAs were classified as up- or down-regulated based on TPM values. A Venn diagram was used to illustrate the overlap of expressed genes among the five experimental groups. To identify differentially expressed genes (DEGs) under different treatments, gene expression profiles were compared across Vv_36 vs. Con, Vv_60 vs. Con, Vh_36 vs. Con, Vh_60 vs. Con, Vv_60 vs. Vv_36, Vh_60 vs. Vh_36, Vh_36 vs. Vv_36, and Vh_60 vs. Vv_60. DEGs were detected using DESeq2 with an adjusted *p*-value (padj) threshold of <0.05. The overall distribution of DEGs across the 15 samples was visualized using column and scatter plots. A heatmap was generated to represent the hierarchical clustering of DEGs based on log_10_(TPM + 1) values, with a specific example provided for the comparison Vv_36 vs. Con. Functional enrichment analysis was performed on DEGs from the Vh_36 vs. Vv_36 and Vh_60 vs. Vv_60. Gene Ontology (GO) term enrichment was analyzed using GOseq (version 1.34.1), which implements a Wallenius non-central hypergeometric distribution model. Kyoto Encyclopedia of Genes and Genomes (KEGG) pathway enrichment was assessed with KOBAS version 2.0 [89] via a hypergeometric test.

### 4.6. QRT-PCR Validation

To validate the RNA-seq results, we selected 13 genes exhibiting a range of log_2_(fold change) (log_2_FC) values in TPM for further confirmation by quantitative real-time PCR (qRT-PCR). These genes were examined across four comparison groups: Vh_36 vs. Con, Vv_36 vs. Con, Vh_60 vs. Con, and Vv_60 vs. Con. QRT-PCR was performed using the RealUniversal Color PreMix (SYBR Green, Tiangen, Beijing, China) on a LightCycler^®^ 480 instrument (Roche, Wilmington, MA, USA). First-strand cDNA synthesized from the RNA samples served as the template. Gene-specific primers were designed for each of the 13 target genes (Table 5), with β-actin used as an internal reference gene for normalization. Sample preparation and amplification procedures followed the protocol described by He et al. [14]. The amplification program consisted of 45 cycles of 95 °C for 10 s, 60 °C for 15 s, and 72 °C for 20 s. Each sample was run in triplicate. The relative expression levels of the target genes were calculated using the 2^−ΔΔCT^ method, expressed as log_2_(2^−ΔΔCT^) = −ΔΔCT. These qRT-PCR results were then compared with the log_2_FC values obtained from the RNA-seq analysis for the same four comparisons to assess consistency between the two methods.

### 4.7. Alternative Splicing Events Analysis

To identify differential alternative splicing (DAS) events, the quality of paired-end sequencing data was first assessed using FastQC v0.10.1. High-quality reads were then aligned to the European eel reference genome (assembly GCF_013347855.1) using HISAT2 2.0.5. Alternative splicing analysis was performed with rMATS v4.0.1 to quantify inclusion levels and detect five types of AS events: skipped exon (SE), retained intron (RI), alternative 3′ splice site (A3SS), alternative 5′ splice site (A5SS), and mutually exclusive exons (MXE). Read length was set to 150 bp for all analyses. Differential AS between *V. harveyi* and *V. vulnificus* infection groups (Vh_36 vs. Vv_36 and Vh_60 vs. Vv_60) was assessed using a likelihood ratio test, with multiple testing correction applied to obtain false discovery rate (FDR) values. Events with FDR < 0.05 were considered significant DAS events. Subsequently, all DAS genes (DASs), DEGs, and their intersections in the two comparisons were subjected to further annotation and analysis, including Venn diagrams, expression profiling, heatmap clustering, and functional enrichment analysis based on GO and KEGG databases.

### 4.8. QPCR Verification of Alternative Splicing Events

To validate the exon-skipping (SE) events identified in the transcriptome analysis, qPCR was performed for three candidate SE events. Specific primers were designed to amplify both the long isoform (containing the alternative exon) and the short isoform (lacking the exon); primer sequences are provided in Table 6. qPCR was carried out using a LightCycler^®^ 480 system (Roche, USA) with RealUniversal Color PreMix (SYBR Green, Tiangen, Beijing, China). Each reaction was run in triplicate under the following cycling conditions: 45 cycles of 95 °C for 10 s, 54 °C for 15 s, and 72 °C for 10 s. A dissociation curve analysis was included to confirm amplification specificity. The expression level of each isoform was calculated using the 2^–ΔCt^ method, where ΔCt = Ct(isoform) − Ct(β-actin). β-actin was used as the internal reference control (primers as in Table 6). The Percent Spliced In (PSI) value was then determined as follows: PSI = Expression level of the longer isoform/(Expression level of the longer isoform + Expression level of the shorter isoform). PSI values were compared across the three experimental groups to assess differential splicing patterns.

### 4.9. Protein–Protein Interaction Network of Proteins Encoded by DAS Genes

The network of 466 cross DAS-encoded proteins with the combined score higher than 0.7, between 2 comparisons of Vh_36 vs. Vv_36 and Vh_60 vs. Vv_60, was constructed to indicate the protein–protein interaction by Cytoscape software (version 3.9.1) to find the essential DASs of host difference in immune response to *V. harveyi* HA_1 and *V. vulnificus* FJ_4 infection.

### 4.10. Statistical Analysis

Fluorescent quantification PCR data were analyzed using the 2^−ΔΔCt^ method for mRNA-seq and the 2^−ΔCt^ method for the splice isoform verification, and graphs were generated using the software Origin 2017.

## Figures and Tables

**Table 1:** Results of RNA sequencing data of 15 samples.

Samples	Raw Reads	Clean Reads	Error Rate (%)	Q20 (%)	Q30 (%)	GC Content (%)
Con_0_1	50,874,554	50,146,920	0.0265	97.38	92.91	50.60
Con_0_2	45,950,964	45,229,786	0.0267	97.31	92.74	50.90
Con_0_3	47,524,136	46,814,670	0.0266	97.36	92.80	50.59
Vv_36_1	71,776,048	69,105,472	0.0243	98.21	94.99	52.19
Vv_36_2	62,302,708	60,657,518	0.0242	98.28	95.14	52.04
Vv_36_3	62,143,894	60,086,848	0.0242	98.27	95.17	52.36
Vh_36_1	46,999,710	46,238,298	0.0268	97.26	92.60	51.83
Vh_36_2	48,078,512	47,406,682	0.0263	97.47	93.08	51.52
Vh_36_3	44,869,076	44,249,230	0.0263	97.46	93.08	50.96
Vv_60_1	66,197,906	63,080,504	0.0242	98.24	95.12	52.29
Vv_60_2	63,084,782	60,768,442	0.0244	98.19	95.01	52.01
Vv_60_3	63,182,062	61,359,548	0.0242	98.24	95.10	52.41
Vh_60_1	43,751,960	43,172,338	0.0262	97.48	93.12	51.02
Vh_60_2	46,005,832	45,378,744	0.0262	97.48	93.13	51.31
Vh_60_3	44,664,684	44,013,716	0.0267	97.30	92.69	50.66

Q20 (%), Q30 (%): Quality assessment of the sequencing data after quality control. Q20 and Q30 refer to the percentage of bases with sequencing quality scores of 99% and 99.9% or higher, respectively, relative to the total bases. Generally, Q20 should be above 85%, and Q30 above 80%; GC content (%): The percentage of G and C bases relative to the total bases in the data after quality control.

**Table 2:** Mapping rates between the reads and referenced genome of *Anguilla anguilla*.

Sample	Total Reads	Total Mapped	Multiple Mapped	Uniquely Mapped
Con_0_1	50,146,920	41,792,062 (83.34%)	1,493,400 (2.98%)	40,298,662 (80.36%)
Con_0_2	45,229,786	37,877,970 (83.75%)	1,262,581 (2.79%)	36,615,389 (80.95%)
Con_0_3	46,814,670	38,945,320 (83.19%)	1,355,266 (2.89%)	37,590,054 (80.30%)
Vv_36_1	69,105,472	60,326,219 (87.30%)	4,028,723 (5.83%)	56,297,496 (81.47%)
Vv_36_2	60,657,518	52,353,692 (86.31%)	2,953,420 (4.87%)	49,400,272 (81.44%)
Vv_36_3	60,086,848	51,726,410 (86.09%)	2,902,755 (4.83%)	48,823,655 (81.26%)
Vh_36_1	46,238,298	39,785,883 (86.05%)	1,453,818 (3.14%)	38,332,065 (82.9%)
Vh_36_2	47,406,682	40,947,894 (86.38%)	1,359,959 (2.87%)	39,587,935 (83.51%)
Vh_36_3	44,249,230	37,733,480 (85.27%)	1,393,405 (3.15%)	36,340,075 (82.13%)
Vv_60_1	63,080,504	53,992,761 (85.59%)	2,146,641 (3.4%)	51,846,120 (82.19%)
Vv_60_2	60,768,442	51,576,117 (84.87%)	1,862,310 (3.06%)	49,713,807 (81.81%)
Vv_60_3	61,359,548	52,756,353 (85.98%)	2,003,358 (3.26%)	50,752,995 (82.71%)
Vh_60_1	43,172,338	37,080,541 (85.89%)	1,204,051 (2.79%)	35,876,490 (83.1%)
Vh_60_2	45,378,744	38,760,833 (85.42%)	1,151,811 (2.54%)	37,609,022 (82.88%)
Vh_60_3	44,013,716	36,931,235 (83.91%)	1,275,855 (2.9%)	35,655,380 (81.01%)

**Table 3:** DEGs involved in at least 7 of 15 enriched KEGG pathways of Vh_36 vs. Vv36.

Gene Name	Gene Description	Pathways	Log_2_FC
*loc118227146*	permeability factor 2-like	7	2.99
*loc118229166*	proto-oncogene c-Fos-like	7	1.78
*loc118223964*	C-X-C motif chemokine 10-like	8	1.75
*loc118221018*	RAC-gamma serine/threonine-protein kinase-like	9	1.38
*loc118207102*	interleukin-1 beta-like, transcript variant X2	10	3.31
*loc118234251*	interleukin-6-like	10	3.42
*loc118228785*	NF-kappa-B inhibitor alpha-like	10	1.04
*nfkbiab*	nuclear factor of kappa light polypeptide gene enhancer in B-cells inhibitor, alpha b	10	1.36
*loc118237077*	transcription factor AP-1-like, transcript variant X1	10	1.79
*june*	JunE proto-oncogene, AP-1 transcription factor subunit	10	2.97
*tnfa*	tumor necrosis factor a	11	3.51
*tnfb*	tumor necrosis factor b	11	2.84

**Table 4:** Number of the alternative splicing/differential alternative splicing (AS/DAS) events and AS/DAS genes identified from each chromosome in Vh_36 vs. Vv_36 and Vh_60 vs. Vv_60 post the challenge of Vh and Vv in American eels (*Anguilla rostrata*).

Chromosome Number	Total AS Events	Total AS Genes	Average AS Events/Genes	Total DAS Events	Total DAS Genes	Average DAS Events/Genes
chrNC_049201	3506, 3370	1152, 1124	3.04, 3.00	229, 175	144, 127	1.59, 1.38
chrNC_049202	3146, 3077	1005, 1027	3.13, 3.00	205, 144	155, 110	1.32, 1.31
chrNC_049203	2798, 2575	876, 856	3.19, 3.01	198, 127	131, 82	1.51, 1.55
chrNC_049204	2832, 2738	857, 849	3.30, 3.22	172, 126	117, 88	1.47, 1.43
chrNC_049205	1999, 2015	681, 671	2.94, 3.00	135, 88	90, 59	1.50, 1.49
chrNC_049206	2418, 2187	788, 745	3.07, 2.94	134, 77	96, 58	1.40, 1.33
chrNC_049207	1917, 1890	646, 647	2.97, 2.92	107, 67	81, 54	1.32, 1.24
chrNC_049208	2294, 2181	775, 756	2.96, 2.88	138, 103	110, 75	1.25, 1.37
chrNC_049209	2346, 2242	745, 730	3.15, 3.07	136, 107	98, 77	1.39, 1.39
chrNC_049210	1744, 1740	630, 639	2.77, 2.72	91, 73	74, 59	1.23, 1.24
chrNC_049211	1957, 1862	618, 610	3.17, 3.05	114, 87	71, 67	1.61, 1.3
chrNC_049212	1521, 1502	491, 491	3.10, 3.06	63, 53	44, 41	1.43, 1.29
chrNC_049213	1827, 1855	578, 582	3.16, 3.19	115, 64	70, 44	1.64, 1.45
chrNC_049214	1954, 1864	554, 531	3.53, 3.51	131, 104	78, 66	1.68, 1.58
chrNC_049215	1312, 1372	427, 438	3.07, 3.13	73, 71	48, 44	1.52, 1.61
chrNC_049216	1309, 1208	439, 420	2.98, 2.88	65, 48	48, 39	1.35, 1.23
chrNC_049217	1777, 1779	545, 529	3.26, 3.36	105, 97	60, 60	1.75, 1.62
chrNC_049218	1058, 1015	379, 369	2.79, 2.75	59, 44	42, 37	1.40, 1.19
chrNC_049219	917, 974	296, 302	3.10, 3.23	47, 42	30, 30	1.57, 1.4
total	38,632, 37,446	12482, 12316	3.10, 3.04	2317, 1697	1579, 1214	1.46, 1.39

Note: Two numbers separated by commas indicate the data of Vh_36 vs. Vv_36 and Vh_60 vs. Vv_60.

**Table 5:** Genes and primers of qRT-PCR validation.

Transcript_id	Gene Name	Gene Description	Primers (5′-3′)
rna-XM_035397752.1	*loc118216518*	β-actin(housekeeping)	F-AATCCACGAGACCACCTTCAA R-GTTGGCGTACAGGTCCTTACG
rna-XM_035421149.1	*ccl34a.3*	chemokine (C-C motif) ligand 34a, duplicate 3	F: CTGCTGCAAAGAGGTCTCCA R: GCTGCATTTCTCCCCCTCTT
rna-XM_035406820.1	*ccr7*	chemokine (C-C motif) receptor 7	F: ACCTGCTGGTGATGCTAACC R: AACACCCACTTGTCAAGGCA
rna-XM_035410631.1	*cd74a*	CD74 molecule, major histocompatibility complex	F: TTCCTGGTGTGCATGCTCAT R: TTCACGAGCGGCTTCTTCTT
rna-XM_035435945.1	*ier3*	immediate early response 3	F: TTTCGAGCAGGTTCCTGTCC R: ATCTGCAGGAACACCACGAG
rna-XM_035391350.1	*il7r*	interleukin 7 receptor	F: CAAGTGCTGAAAACACCGGG R: CAGAAATGAGTCGGCAGGGT
rna-XM_035394107.1	*marco*	macrophage receptor with collagenous structure	F: GAGGCACCGGAATAGTCGAG R: TGGTCCTGAGCCCATATGGA
rna-XM_035388377.1	*tktb*	transketolase b	F: GCAGACCCAGACCTTTTCGA R: TGCGTCGATGTCAAAGGTCA
rna-XM_035387337.1	*loc118210870*	tumor necrosis factor receptor superfamily member 9-like	F: CACACCTGCCCCAAAACATG R: CCAAGAGCCGTACACCACTT
rna-XM_035389795.1	*loc118212156*	lysozyme C-like	F: GCATTTGGTGGCTTGTGGAC R: CTTCTCCAGCTTGCCCTCTC
rna-XM_035398056.1	*loc118216669*	ferritin, middle subunit-like	F: GGACTCTCACTTCTGCCACC R: CCACCGGCAGATCGTTGATA
rna-XM_035425000.1	*loc118231298*	H-2 class II histocompatibility antigen, A-U alpha chain-like	F: GGTCTGTTCACCTGCTTCGA R: TGTCTCCCAGGGGCTGAATA
MSTRG.16029.33	*loc118231311*	H-2 class II histocompatibility antigen, E-S beta chain-like	F: GGTCTGTTCACCTGCTTCGA R: TGTCTCCCAGGGGCTGAATA
rna-XM_035431152.1	*loc118234548*	biotinidase-like, transcript variant X1	F: TTCGCCTCCTACTACCAGCT R: TAGGCTTTCCCTTGAGCTGC

**Table 6:** Primers of qRT-PCR validation for three exon-skipping events.

AS ID	Gene Name	Gene Description	Chromosome	Primers (5′-3′)
SE_4646	*loc118208085*	serine-aspartate repeat-containing protein F-like, transcript variant X7	NC_049211.1	Fi-ggcttctctggaggtggttt Ri-ctgaaaatgcatcagttaataagtc 136 bp Fs-caagcagcagcacaggaaaatgg Rs = Ri 62 bp
SE_10321	*tnfsf13*	TNF superfamily member 13, transcript variant X3	NC_049209.1	Fi-cccctctcctcacactcatacg Ri-agtaggaccctccagtcgac 126 bp Fs-cagctcggtaaacgatgaggaggac Rs = Ri 120 bp
SE_5690	*znf*3*41*	zinc finger protein 341, transcript variant X4	NC_049211.1	Fi-tctactggaacaacagccgc Ri-cttcaaatattgcctgcgcca 104 bp Fs-actatacaacctatggggctaaatctacagag Rs = Ri 105 bp

SE: exon skipping; i: the exon that is included; s: the exon that is skipped.

## Data Availability

All 15 files of transcriptome RNA-seq data (SRA) have been uploaded to NCBI, and the BioProject ID is PRJNA988445.

## Supplementary Materials

The following supporting information can be downloaded at: https://www.mdpi.com/article/10.3390/ijms262411763/s1.
